# International study of perceived neighbourhood environmental attributes and Body Mass Index: IPEN Adult study in 12 countries

**DOI:** 10.1186/s12966-015-0228-y

**Published:** 2015-05-16

**Authors:** Ilse De Bourdeaudhuij, Delfien Van Dyck, Deborah Salvo, Rachel Davey, Rodrigo S. Reis, Grant Schofield, Olga L. Sarmiento, Josef Mitas, Lars Breum Christiansen, Duncan MacFarlane, Takemi Sugiyama, Ines Aguinaga-Ontoso, Neville Owen, Terry L. Conway, James F. Sallis, Ester Cerin

**Affiliations:** Department of Movement and Sport Sciences, Ghent University, Ghent, Belgium; Research Foundation – Flanders (FWO), Brussels, Belgium; Center for Nutrition and Health Research, National Institute of Public Health, Cuernavaca, Mexico; Centre for Research & Action in Public Health, Faculty of Health, Canberra University, Canberra, Australia; School of Health and Biosciences, Pontificia Universidade Catolica do Parana, Curitiba, Brazil; Department of Physical Education, Federal University of Parana, Curitiba, Brazil; Human Potential Centre, AUT University, Auckland, New Zealand; Department of Public Health, School of Medicine, Universidad de los Andes, Bogotá, Colombia; Institute of Active Lifestyle, Faculty of Physical Culture, Palacký Universitsy, Olomouc, Czech Republic; Department of Sports Science and Clinical Biomechanics, University of Southern Denmark, Odense, Denmark; Institute of Human Performance, The University of Hong Kong, Hong Kong, China; School of Population Health, University of South Australia, Adelaide, Australia; Department of Health Sciences, Public University of Navarra, Pamplona, Navarra Spain; Baker IDI Heart & Diabetes Institute, Melbourne, VIC Australia; Department of Family and Preventive Medicine, University of California, San Diego, USA; Centre of Physical Activity and Nutrition Research, School of Exercise and Nutrition Sciences, Deakin University, Melbourne, Australia

**Keywords:** Weight status, Built environment, International, Pooled data

## Abstract

**Background:**

Ecological models of health behaviour are an important conceptual framework to address the multiple correlates of obesity. Several single-country studies previously examined the relationship between the built environment and obesity in adults, but results are very diverse. An important reason for these mixed results is the limited variability in built environments in these single-country studies. Therefore, the aim of this study was to examine associations between perceived neighbourhood built environmental attributes and BMI/weight status in a multi-country study including 12 environmentally and culturally diverse countries.

**Methods:**

A multi-site cross-sectional study was conducted in 17 cities (study sites) across 12 countries (Australia, Belgium, Brazil, China, Colombia, Czech Republic, Denmark, Mexico, New Zealand, Spain, the UK and USA). Participants (n = 14222, 18–66 years) self-reported perceived neighbourhood environmental attributes. Height and weight were self-reported in eight countries, and measured in person in four countries.

**Results:**

Three environmental attributes were associated with BMI or weight status in pooled data from 12 countries. Safety from traffic was the most robust correlate, suggesting that creating safe routes for walking/cycling by reducing the speed and volume of traffic might have a positive impact upon weight status/BMI across various geographical locations. Close proximity to several local destinations was associated with BMI across all countries, suggesting compact neighbourhoods with more places to walk related to lower BMI. Safety from crime showed a curvilinear relationship with BMI, with especially poor crime safety being related to higher BMI.

**Conclusions:**

Environmental interventions involving these three attributes appear to have international relevance and focusing on these might have implications for tackling overweight/obesity.

## Background

Overweight and obesity are important health problems in developed and developing countries [1]. The ecological model has been increasingly used as a conceptual framework to address obesity at multiple levels (individual, inter-personal/social environment, built environment, and policies) [2]. The premise is that by reducing the obesogenicity of the environment, the obesity problem can be tackled on a larger scale and reach a wider population.

In the past decade, built environmental factors were mainly studied in relation to physical activity (PA). Results showed a consistent relationship between PA (active transport and recreational walking) and neighbourhood walkability in adults [3–6].

Investigating the relationship between characteristics of the built environment and adiposity (e.g., BMI, overweight/obesity) has often been secondary to investigating their relationships with PA or diet, and findings are less clear. A recent review of reviews [3] identified 8 review articles that examined the relationship between the built environment and overweight/obesity in adults [7–14]. The findings of these reviews are very diverse. A review focusing on smart growth factors (e.g., higher density, diverse land use) showed that few studies reported significant associations with adiposity measures [8]. In contrast, another review showed lower average BMI in neighbourhoods with higher perceived mixed land-use, improved walkability, and better access to facilities [10]. Also, significant associations between some aspects of the objectively measured built environment (e.g. residential density, street connectivity, greenery, access to destinations) and obesity were observed in 84 % of the studies [7].

Although some of the apparent discrepancies could be due to differences in methods, the main issue that may explain this diversity in results is the limited variability. Almost all studies of relationships between the built environment and overweight/obesity have been conducted within single countries and usually within a single city. Limited variability in built environments in these studies might be a reason for non-significant associations. However, a few single-country studies were explicitly designed to maximize built environment variability, and most of those studies are included in present analyses of pooled international data (NQLS, BEPAS, PLACE). Combining data from environmentally and culturally different contexts may help to better understand how neighbourhood built environments are related to residents’ adiposity.

The present study's purpose was to examine the strength, direction, and shape of the associations between perceived neighbourhood built environmental attributes and adiposity measures using pooled data from the 12 countries participating in the International Physical Activity and the Environment Network (IPEN) Adult study. Variation in the associations by study sites was also explored.

## Methods

### Study design

The IPEN Adult study is an observational, epidemiologic, multi-country, cross-sectional study, including 17 city-regions (hereafter, sites) within 12 countries: Australia (Adelaide), Belgium (Ghent), Brazil (Curitiba), China (Hong Kong), Colombia (Bogota), Czech Republic (Olomouc, Hradec Kralove), Denmark (Aarhus), Mexico (Cuernavaca), New Zealand (North Shore, Waitakere, Wellington, Christchurch), Spain (Pamplona), the UK (Stoke-on-Trent) and USA (Seattle, Baltimore).

Participants were mostly recruited from neighbourhoods chosen to maximize variance in neighbourhood walkability and socio-economic status (SES) [15]. For neighbourhood selection, all countries, except Spain, used an objective GIS-based neighbourhood walkability index [16, 17], with each country’s selection strategy reported elsewhere [15]. Administrative units were ranked by walkability index and median household income (neighbourhood-level SES), and approximately equal numbers of neighbourhoods were selected to represent four categories: high-walkable/high-SES, high-walkable/low-SES, low-walkable/high-SES, and low-walkable/low-SES.

### Participant recruitment

Adults living in the selected neighbourhoods were systematically contacted over 2002–2011. Four countries used phone/mail/online surveys; seven countries delivered study materials directly to participants; whilst Hong Kong used intercept interviews. Although ages ranged from 16–94, we limited the present analyses to 18–66 years.

### Quality control

All investigators completed the San Diego State University IRB training, and met the NIH Fogarty International Center and their local ethics requirements. All participants provided informed consent. Confidentiality for pooled data maintained using only identification codes before transmitting data to the IPEN Coordinating Center, where it was processed to maximize completeness, comparability, and consistent-coding.

### Measures

#### Body mass index (BMI) and weight status

To calculate BMI, participants reported their height and weight in eight countries or were measured objectively in Brazil, Mexico, New Zealand, and UK. Self-reported and objectively measured BMI are highly correlated, and BMI is a proxy measure for adiposity in large-scale studies [18]. Participants were then categorised into normal weight and overweight/obese (BMI ≥ 25 kg/m^2^).

#### Perceptions of neighbourhood built environmental attributes

The Neighbourhood Environment Walkability Scale (NEWS) assesses perceived neighbourhood attributes believed to be related to physical activity and reflects key exposure measures in the IPEN study [19, 20]. As IPEN countries used adapted versions, extensive item comparisons (made by at least 2 independent raters) and confirmatory factor analyses were completed. Cerin et al. [21] reported the following 10 NEWS subscales that can be used for the IPEN multi-country pooled analyses: (1) Residential density; (2) Land use mix–access; (3) Land use mix–diversity; (4) Street connectivity; (5) Infrastructure and safety for walking; (6) Aesthetics; (7) Safety from traffic; (8) Safety from crime; (9) Streets having few cul-de-sacs; and (10) No major physical barriers to walking.

The *Residential density* subscale is a weighted sum of items reflecting perceived density of housing, ranging from predominantly single-family dwellings to high-rise buildings with more than 20 stories. *Land use mix–diversity* reflects average perceived walking proximity (i.e., average of five-point ratings ranging from ≤5 minute walk to 30+ minute walk) from home to 9 types of destinations (e.g., supermarket, school, transit stop, and other stores and services). The remaining eight scales are average ratings of items answered on a four-point Likert scale (1 = strongly disagree to 4 = strongly agree). Scoring details are described elsewhere (21). Additionally, for the current study, a composite perceived walkability index was computed by summing the z-scores of all perceived neighbourhood walkability attributes.

#### Socio-demographic characteristics

Age, gender, educational level, and marital status were assessed and included as covariates in all statistical models. Education level was categorized into having ‘university degree’, ‘high school diploma’ and ‘less than high school diploma’. Marital status was dichotomized into married/de facto or not-married.

### Data analytic plan

Descriptive statistics were computed for the whole sample and by study site for all variables. There were 8.2 % cases with missing data on at least one variable. Consequently, ten imputed datasets were created for the main regression analyses (see below) as recommended by Rubin [22] and van Buuren [23]. Multiple imputations were performed using chained equations (MICE) [23] accounting for the two-stage stratified sampling strategy employed in each study site (see Methods section).

We examined associations of built environment with body mass index (kg/m^2^) and weight status (normal vs. overweight/obese) using generalized additive mixed models (GAMMs) [24]. A set of GAMMs used binomial variance and logit link functions, appropriate for dichotomous measures of weight status. These models yielded odds ratios of being vs. not being overweight/obese. BMI was modelled using GAMMs with the residual variance proportional to the outcome mean (variance corresponding to the cube of the mean) and with a logarithmic link function [24]. The reported antilogarithms of the regression coefficient estimates of these two GAMMs represent the proportional increase in BMI associated with a unit increase in the predictor.

A main-effect set of GAMMs estimated the dose–response relationships of all perceived environmental attributes and, in separate models, of the composite walkability index with the continuous and categorical BMI outcome variables, adjusting for study site, socio-demographic covariates, and the design variable administrative-unit-level socio-economic status (low versus high), as well as accounting for dependency in error terms due to clustering of participants sampled from pre-selected administrative units. Curvilinear relationships of environmental attributes with outcomes were estimated using non-parametric smooth terms, which were modelled using thin-plate splines [24]. Separate GAMMs were run to estimate environmental attributes by study site interaction effects. The significance of interaction effects was evaluated by comparing QAIC values of models with and without a specific interaction term (the model with the smaller QAIC was preferred). Significant interaction effects were probed by computing site-specific associations using linear functions. Finally, the proportion of city-level variance of probability of being overweight/obese and BMI explained by perceived environmental attributes was computed. All analyses were conducted in R [25] using the packages ‘car’ [26], ‘mgcv’ [24], ‘gmodels’ [27], and ‘mice’ [28].

## Results

**Fig. 1:**
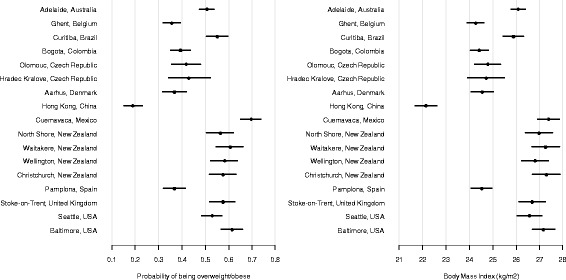
Between-site differences in probability of being overweight/obese and average body mass index (kg/m^2^) adjusted for socio-demographic characteristics and perceived environmental attributes

Table 1 shows descriptive statistics for each study site including socio-demographic characteristics, weight status, and BMI. The total sample consisted of 14,222 participants; 57 % women, 60 % lived with a partner, 44 % with a college/university degree, and 74 % worked. The mean age was 42 years (SD = 12.8). After adjusting for environmental predictors and socio-demographic covariates, Cuernavaca (Mexico) had the highest and Hong Kong (China) the lowest prevalence of overweight/obesity (Fig. 1). In general, the average BMI and prevalence of overweight/obesity were lower in European sites (except for Stoke-on-Trent, UK) and higher in North and South American (except for Bogota, Colombia), Australian and New Zealand sites (Fig. 1).

**Table 1:** Overall and site-specific sample characteristics: socio-demographics, body mass index (BMI), and weight status

	ALL SITES	AUS	BEL	BRA	COL	CZE		DEN	HK	MEX	NZ				ESP	UK	USA	
						Site A	Site B		Studies 1 and 2		Site C	Site D	Site E	Site F			Site G	Site H
Overall N^a^	14222	2650	1166	697	963	330	167	642	970	677	511	512	496	495	904	843	1287	912
Mean age (SD)	42 (12.8)	44 (12.3)	43 (12.6)	41 (13.2)	40 (13.7)	38 (14.7)	34 (13.1)	39 (13.9)	43 (12.3)	42 (12.6)	41 (11.8)	41 (11.8)	39 (12.6)	42 (12.6)	39 (14.2)	43 (13.3)	44 (11.0)	47 (10.7)
Gender, *%men*	43	36	48	47	36	37	40	43	39	45	36	39	49	44	45	44	55	48
Education, *%*																		
*Less than HS*	18	24	4	29	36	22	17	8	40	43	4	5	1	11	7	34	1	2
*HS graduate*	38	30	35	32	42	46	57	44	23	29	58	64	47	57	35	52	36	30
*College or more*	44	46	61	39	22	32	26	48	37	28	38	31	52	32	58	14	63	68
Work status, *% working*	74	71	80	78	58	77	84	75	63	72	78	84	87	80	72	64	81	83
Marital status, *% couple*	60	57	73	58	53	58	47	65	59	65	70	74	57	55	53	45	63	60
Mean BMI km/m^2^ (SD)	25.7 (5.2)	26.2 (5.9)	24.3 (3.9)	26.1 (4.5)	24.7 (4.1)	24.4 (3.8)	24.0 (3.4)	24.1 (3.7)	22.1 (3.3)	28.0 (5.0)	26.9 (5.7)	27.4 (5.6)	26.4 (5.1)	27.4 (6.1)	23.9 (3.6)	27.6 (5.6)	26.6 (5.5)	27.2 (5.7)
Weight status, %																		
*Underweight*	3	2	3	2	4	3	3	2	10	1	2	1	0	1	3	1	1	1
*Normal*	49	47	60	43	56	59	61	65	71	27	42	37	44	40	65	36	45	37
*Overweight*	31	32	29	37	29	29	30	27	16	41	32	37	39	32	26	35	34	40
*Obese*	17	19	8	18	11	9	6	6	3	31	24	25	17	27	6	28	20	22

*Notes:* ^a^N for some variables is reduced due to missing data. Site A: Olomouc, B: Hradec Kralove, C: North Shore, D: Waitakere, E: Wellington, F: Christchurch, G: Seattle, H: Baltimore

Missing data: age (1.4 %), gender (0.3 %), education (1,2 %), work status (0.4 %), marital status (1.2 %), BMI (3.1 %)

Average perceived residential density was the lowest in three out of four study sites located in New Zealand, and the highest in Hong Kong, followed by Pamplona (Spain) and Curitiba (Brazil) (Table 2). All study sites had relatively high average scores on land use mix. Pamplona (Spain) had the highest scores on seven out of ten perceived environmental characteristics, while Cuernavaca (Mexico) and Waitakere (New Zealand) had one of the lowest average scores on five characteristics. The composite walkability indices of the study sites in New Zealand, Cuernavaca (Mexico), Bogota (Colombia) and Ghent (Belgium) were relatively low, while those of Hong Kong (China), Aarhus (Denmark) and Pamplona (Spain) were high.

**Table 2:** Overall and site-specific perceived environmental scores [mean (SD)], all scores are in the positive direction, higher scores meaning more agreement with the attribute

	All SITES	AUS	BEL	BRA	COL	CZ		DEN	HK	MEX	NZ				ESP	UK	USA	
						Site A	Site B		Studies 1 and 2		Site C	Site D	Site E	Site F			Site G	Site H
Overall N^a^	14222	2650	1166	697	963	330	167	642	970	677	511	512	496	495	904	843	1287	912
Residential density	88 (133)	36 (41)	84 (73)	100 (123)	77 (82)	91 (70)	92 (70)	86 (65)	414 (240)	38 (41)	29 (47)	18 (26)	49 (68)	22 (25)	200 (104)	40 (41)	39 (57)	60 (79)
Land use mix – diversity (9 destination types)	3.9 (0.7)	3.8 (0.7)	3.6 (0.9)	4.1 (0.5)	4.3 (0.5)	3.9 (0.6)	3.9 (0.7)	4.2 (0.6)	4.0 (0.8)	3.7 (0.6)	3.8 (0.7)	3.6 (0.7)	4.1 (0.6)	3.9 (0.6)	4.6 (0.4)	3.6 (0.7)	3.8 (0.8)	3.6 (0.9)
Land use mix - access	3.4 (0.7)	3.5 (0.7)	3.3 (0.6)	3.7 (0.5)	3.4 (0.5)	3.4 (0.7)	3.4 (0.7)	3.6 (0.6)	3.3 (0.8)	3.3 (0.5)	3.2 (0.6)	3.1 (0.5)	3.4 (0.5)	3.3 (0.5)	3.7 (0.5)	3.3 (0.8)	3.2 (0.8)	3.0 (0.8)
Connectivity	3.0 (0.7)	2.8 (0.9)	2.7 (0.7)	3.3 (0.7)	3.2 (0.5)	3.0 (0.7)	2.9 (0.6)	3.0 (0.6)	3.0 (0.8)	2.9 (0.5)	2.7 (0.5)	2.7 (0.4)	2.8 (0.5)	3.0 (0.5)	3.2 (0.7)	3.1 (0.7)	3.0 (0.8)	3.0 (0.8)
Infrastructure and safety	3.0 (0.6)	3.0 (0.6)	2.8 (0.5)	2.8 (0.8)	2.8 (0.5)	3.1 (0.5)	3.2 (0.5)	3.1 (0.5)	3.3 (0.6)	2.6 (0.4)	2.8 (0.3)	2.8 (0.4)	2.9 (0.4)	2.9 (0.4)	3.3 (0.5)	3.1 (0.5)	3.0 (0.6)	3.1 (0.6)
Aesthetics	2.8 (0.7)	2.9 (0.7)	2.5 (0.6)	2.8 (0.8)	2.5 (0.6)	2.4 (0.6)	2.5 (0.6)	2.7 (0.6)	2.7 (0.7)	2.6 (0.5)	2.8 (0.5)	2.8 (0.5)	2.8 (0.5)	2.8 (0.6)	2.8 (0.7)	2.2 (0.8)	3.1 (0.7)	3.1 (0.6)
Safety from traffic	2.6 (0.7)	2.8 (0.8)	2.4 (0.6)	2.4 (0.8)	2.5 (0.5)	2.9 (0.6)	3.1 (0.5)	2.8 (0.5)	2.7 (0.7)	2.4 (0.5)	2.6 (0.5)	2.6 (0.5)	2.8 (0.4)	2.7 (0.5)	2.4 (0.7)	2.5 (0.7)	2.7 (0.7)	2.7 (0.7)
Safety from crime	3.0 (0.8)	3.0 (0.8)	3.1 (0.6)	2.3 (0.5)	2.1 (0.7)	3.2 (0.6)	3.4 (0.6)	3.3 (0.6)	2.8 (1.1)	2.2 (0.5)	3.0 (0.5)	2.9 (0.4)	3.1 (0.4)	2.9 (0.6)	3.5 (0.6)	2.9 (0.8)	3.4 (0.6)	3.4 (0.7)
Few cul-de-sacs	2.8 (1.0)	2.8 (1.1)	3.0 (0.8)	3.0 (1.1)	2.9 (0.8)	2.9 (0.9)	2.9 (0.9)	2.7 (0.9)	2.9 (1.2)	2.6 (0.8)	2.3 (0.7)	2.3 (0.6)	2.5 (0.7)	2.6 (0.8)	3.5 (0.9)	2.4 (1.0)	2.8 (1.1)	2.8 (1.2)
No major barriers	3.3 (0.9)	3.7 (0.7)	3.3 (0.7)	3.1 (1.0)	3.0 (0.7)	3.4 (0.8)	3.5 (0.8)	3.7 (0.6)	2.7 (1.2)	2.8 (0.7)	3.3 (0.6)	3.2 (0.6)	3.3 (0.5)	3.5 (0.6)	3.6 (0.8)	3.3 (0.8)	3.2 (1.0)	3.7 (0.6)

*Notes:* ^a^N for some variables is reduced due to missing data, SD: standard deviation. Site A: Olomouc, B: Hradec Kralove, C: North Shore, D: Waitakere, E: Wellington, F: Christchurch, G: Seattle, H: Baltimore

Missing data: residential density (2.4 %), land use mix diversity (0.7 %), land use mix access (0.7 %), connectivity (0.7 %), infrastructure and safety (0.5 %), aesthetics (0.6 %), safety from traffic (0.7 %), safety from crime (0.7 %), cul-de-sacs (0.9 %), no major barriers (0.8 %)

Female, younger, single, highly educated participants and those living in higher SES areas were less likely to be overweight/obese (Table 3). When accounting for other perceived environmental attributes, traffic safety was the only attribute negatively related to the odds of being overweight/obese (Table 3). A significant interaction effect of study site by perceived pedestrian infrastructure and safety on the odds of being overweight/obese was observed. Namely, a positive association between pedestrian infrastructure and safety and the odds of being overweight/obese was observed in Hong Kong (China) (OR = 1.45; 95 % CI: 1.07, 1.96; p < .05), a negative association in Adelaide (Australia) (OR = 0.80; 95 % CI: 0.70, 0.94; p < .01), while the other sites showed no significant association.

**Table 3:** Linear and curvilinear associations of socio-demographic and perceived environmental attributes with weight status (normal vs. overweight/obese) and body mass index (kg/m^2^) (N = 14222)

Predictor	Odds of being overweight/obese^a^			Body mass index^b^		
	OR	95 % CI	p	exp(b)	exp(95 % CI)	p
***Socio-demographic***						
Gender (reference: male)						
Female	0.60	0.56, 0.65	<.001	0.97	0.96, 0.98	<.001
Area socio-economic status (reference: low)						
High socio-economic status	0.89	0.80, 0.98	.021	0.98	0.97, 0.99	<.001
Education (reference: less than high school )						
High school graduate	0.99	0.89, 1.11	.903	0.99	0.98, 1.00	.167
College or more	0.69	0.61, 0.78	<.001	0.96	0.95, 0.97	<.001
Working status (reference: not working)						
Working	0.98	0.90, 1.07	.665	0.99	0.99, 1.00	.069
Marital status (reference: single)						
Couple	1.19	1.10, 1.29	<.001	1.00	0.99, 1.01	.291
Age (yrs)	1.60	1.14, 2.26	<.001	1.05	1.01, 1.09	.006
***Perceived environmental attributes***						
Residential density	1.000	0.999, 1.000	.079	1.001	0.999, 1.003	.138
Land use mix – access	1.07	0.99, 1.15	.078	1.01	0.99, 1.01	.064
Land use mix – diversity (9 destination types)	0.94	0.88, 1.00	.062	0.99	0.99, 1.00	.043
Connectivity	1.00	0.94, 1.05	.902	1.00	0.99, 1.00	.137
Infrastructure and safety	0.98	0.91, 1.06	.595	1.00	0.99, 1.01	.819
Aesthetics	0.96	0.90, 1.03	.287	0.99	0.99, 1.00	.055
Safety from traffic	0.92	0.86, 0.97	.005	0.99	0.99, 1.00	.002
Safety from crime	0.99	0.92, 1.04	.496	0.99	0.99, 1.00	.071
Curvilinear component	-	-	-	F(1.52) = 4.72		.017
Few cul-de-sacs	0.98	0.94, 1.02	.364	1.00	1.00, 1.00	.792
No major barriers	0.97	0.92, 1.02	.177	1.00	0.99, 1.00	.480
Composite walkability score	0.98	0.97, 0.99	.002	0.992	0.88, 0.995	<.001

*Note.* Regression coefficients are adjusted for other perceived environmental characteristics, respondents’ age, gender, marital status, educational attainment, employment status, and administrative-unit (neighborhood) socio-economic status. OR = odds ratio; 95 % CI = 95 % confidence intervals; exp(b) = antilogarithm of regression coefficient; exp(95 % CI) = antilogarithm of confidence intervals; − = not applicable. ^a^generalized additive mixed model (GAMM) with binomial variance and logit link functions. ^b^GAMM base on quasi-likelihood approach with logarithmic link function and variance proportional to the cube of the outcome mean. For these models, exp(b) is to be interpreted as the proportional increase in body mass index associated with a 1 unit increase on the predictor

The composite walkability index was significantly negatively related to the odds of being overweight/obese (OR = 0.98; 95 % CI: 0.97, 0.99; p = .002). The difference in minimum and maximum scores across study sites in the walkability index was nearly 30 units. Thus, the odds of being overweight/obese for a person living in the most walkable neighbourhood were 45 % lower than those of a person living in the least walkable neighbourhood. These effects did not significantly differ across study sites. Perceived environmental attributes explained 11.8 % of site-level variance in the probability of being overweight/obese, corresponding to a standard deviation of 4.7 % in prevalence of overweight/obesity and 28.2 % in prevalence between the lowest and highest ranked study site on perceived neighbourhood attributes.

**Fig. 2:**
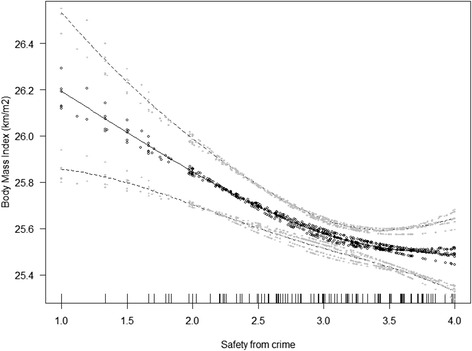
Non-linear relationship between perceived safety from crime and body mass index (kg/m^2^)

BMI was lower in female, higher educated, younger participants, and those living in higher socio-economic status neighbourhoods (Table 3). Higher levels of land use mix–diversity and traffic safety were predictive of lower BMI. A unit increase on each of these two environmental measures was associated with a 1 % decrease in average BMI (Table 3). The association of perceived safety from crime with BMI was negative and curvilinear; BMI decreases as safety increases, but is relatively constant at the higher end of the safety from crime scale (Fig. 2). Study site moderated the associations of BMI with perceived residential density and no major barriers to walking. Specifically, only two study sites showed significant, negative associations between perceived residential density and BMI. These were Adelaide, Australia (OR = 0.9996; 95 % CI: 0.9994, 0.9998; p < 0.001) and Waitakere, New Zealand (OR = 0.9992; 95 % CI: 0.9985, 0.9999; p < 0.05). Negative associations of BMI and no perceived major barriers to walking were observed in Adelaide, Australia (OR = 0.987; 95 % CI: 0.977, 0.998; p < 0.05) and Waitakere, New Zealand only (OR = 0.967; 95 % CI: 0.943, 0.993; p < 0.05).

The composite walkability index was significantly negatively related to BMI (OR = 0.992, 95 % CI = 0.88, 0.995; p < 0.001). Study site did not significantly moderate this association. Perceived environmental attributes explained 5.9 % of city-level variance in BMI, corresponding to a standard deviation of 0.37 kg/m^2^ and 2.24 kg/m^2^ difference between the lowest and highest ranked study site on perceived neighbourhood attributes.

## Discussion

In the pooled analyses including all 12 countries, safety from traffic was the only environmental attribute that was associated in the expected direction both with lower odds of being overweight/obese, and lower BMI. Participants’ BMI was linearly related to land use mix-diversity and curvilinearly associated with safety from crime. It should be noted that these three environmental attributes (safety from traffic, land use mix-diversity, and safety from crime) show a consistent relationship with the outcome measures in 12 environmentally and culturally diverse countries. The linear relationships found for land use mix-diversity and traffic safety in these countries suggest that the closer the perceived walking proximity to destinations (supermarket, grocery, post office, transit stop, restaurant, park, gym, school), and the safer it is to reach these destinations (no heavy traffic, slow traffic speeds), the lower the BMI of the inhabitants of the regions is. Previous research showed a consistent relationship between land use mix-diversity and physical activity [3], while associations between traffic safety and physical activity are less consistent [29, 30]. Because land use mix and traffic safety have been related to more walking for transportation purposes, transport-walking is a potential mediator of the observed association between these two environmental attributes and body mass. However, clear and consistent evidence on the relationships of these environmental attributes with body mass is lacking. Black et al. [10] concluded from their review of 37 studies that a lower average BMI seemed to be related to higher mixed land use, improved walkability, better access to facilities, and low perceived hazards. However, their conclusion was derived by examining mainly studies conducted in the US. The present study supports the importance of diverse land use (the presence of destinations nearby) and neighbourhood safety in residents’ weight status through examining the large, international data with a wider variability in environmental factors and obesity levels.

The present study makes an important contribution by investigating the shape of the relationship between built environment variables and BMI. The curvilinear relationship with perceived safety from crime can be understood as a threshold effect. The curvilinear association suggests that in neighbourhoods with low perceived crime safety, a small increase in perception of crime safety could be associated with a decrease in BMI. However, in relatively safe neighbourhoods, few additional effects on BMI could be expected from further increasing the perceived safety from crime. Thanks to the large between-country variance in built environmental characteristics and weight status, the present findings can give further insights into the often conflicting results found in the past for the relationship between perceived safety from crime and physical activity/body mass [30, 31]. Especially in countries that have relatively high crime rates, lower perceived safety from crime at night seems to be an important feature related to higher BMI [32].

Very few interactions by study site were revealed in the analyses, suggesting that associations were similar across countries. A site-specific result was found for perceived pedestrian infrastructure and safety on the odds of being overweight/obese. A positive association was found in Hong Kong, a negative association in Australia, and nonsignificant associations in all other countries. Another site-specific result was observed for residential density and no major barriers on BMI. Negative associations between BMI and both environmental attributes were only found in Australia and New Zealand. These results suggest that the relationship between perceived built environmental attributes and BMI/overweight might be somewhat stronger in Australia compared to other countries. Previous studies on the relationship between the built environment and body mass in Australian adults showed mixed results. A recent study by Christian et al. [33] showed almost no relationship of perceived environmental factors with BMI, while other studies showed significant associations [34, 35]. Future research is needed to further clarify this finding, in order to be able to decide whether similar intervention strategies could possibly have generalizable effects across countries.

Despite the consistent association of BMI with land-use mix diversity, safety from traffic and safety from crime in the present study, the other perceived environmental attributes that were examined, such as residential density, land use mix access (to shops and services), street connectivity, pedestrian/bicycling infrastructure, aesthetics, absence of cul-de-sacs, and other major barriers were not associated with BMI or weight status across the countries. As discussed in the previously cited review [3], neighbourhood environmental attributes that were found to be consistently related to PA do not necessarily have direct associations with BMI. However, the composite walkability index including the sum of all perceived environmental attributes was significantly negatively related to both the odds of being overweight/obese and to BMI. As these effects did not significantly differ across study sites, they show that a combination of more favourable environmental attributes is related to less overweight across the world. It is notable that the composite walkability score had a relatively large effect size. In the case of the odds of overweight/obesity, the most walkable study site had 45 % lower odds than the least walkable site. In contrast, a single item showed a smaller effect size. For instance, the odds of overweight/obesity in the safest site (from crime) was 11 % lower than that of the least safe site. This can be interpreted as showing that multiple environmental attributes may have an accumulated impact on residents’ weight status. Such accumulated environmental influence has been also suggested for physical activity [36]. Future research can investigate the impact of multiple environmental attributes on adults’ adiposity.

The major strength of the present study was the unique pooling of data across 12 countries, to increase the variability in built environment characteristics. Another strength is the use of advanced statistical analyses investigating linear as well as curvilinear relationships. Furthermore, the study used a large sample, including more than 14,000 adults. Limitations included the mix of self-report and objective measures for BMI across countries. This variation in measurement methods could have under- or over-estimated observed associations. A second limitation is the use of standard BMI cut-points in all countries. As no ethnicity information was available, more specific BMI cut-points (eg. Asian specific) could not be used. The use of perceived environmental attributes is sometimes considered to be a limitation. Within the IPEN study, objective GIS-based environmental measures are also available in almost all countries. Previous research has shown that perceived as well as objective measures of the built environment can explain physical activity or BMI [37]. In addition, it is well documented that people’s environment perceptions often differ from objectively-identified walkability measures [38, 39]. The present study focused on the perceived environment related to weight status/BMI, and GIS based measures will be included in future manuscripts dependent on the availability of the measures in subsamples of countries. It was beyond the scope of the present manuscript to study the potential mechanisms through which neighbourhood environmental attributes relate to BMI. Future manuscripts will examine these mechanisms by conducting analyses to examine how multiple measures of physical activity and sedentary behaviours may mediate the relation between built environments and BMI. Diet intake should also be included in future studies in the pathway between environmental attributes and BMI. However, diet was not measured in the IPEN Adult study.

## Conclusion

In summary, three environmental attributes were associated with BMI or weight status in pooled data from 12 countries, over and above the effect of socio-demographic characteristics and neighbourhood-level SES. Safety from traffic was the most robust correlate, which suggests that creating safe routes for walking or cycling by reducing the speed and volume of traffic might contribute to improved weight status and BMI across various geographical locations. Close proximity to several destinations was associated with lower BMI across all countries, suggesting that compact neighbourhoods with more places to walk to could be an important policy goal for controlling obesity internationally. Safety from crime showed a curvilinear relationship with BMI, with the relation of perceived crime safety to lower BMI being more pronounced in areas with poorer perceived safety. This suggests that enhanced efforts to improve neighbourhood safety (through reducing crime or incivilities, or enhancing surveillance) should be targeted mainly in areas with low perceived crime safety, and if effective, could play a role in obesity prevention. Environmental interventions involving these three attributes appear to have international relevance and may help reduce the prevalence of overweight and obesity across the world. To achieve such changes, it will likely be necessary for public health professionals to work with policy makers and practitioners in transportation (to improve traffic safety), urban planning (to ensure mixed use development), and law enforcement (to reduce crime) [40]. Future longitudinal or quasi-experimental studies documenting weight changes in neighbourhoods are needed to build more robust evidence in support of policy changes implied by the findings of this study.
